# Comparative Efficacy of Three Forms of Parenteral Iron

**DOI:** 10.1155/2012/473514

**Published:** 2011-11-22

**Authors:** Richard Dillon, Ibrahim Momoh, Yvonne Francis, Laura Cameron, Claire N. Harrison, Deepti Radia

**Affiliations:** ^1^Department of Haematology, Guy's and St Thomas' NHS Foundation Trust, London SE1 9RT, UK; ^2^Department of Haematology, Kings College Hospital, London SE5 9RS, UK

## Abstract

Intravenous iron therapy is a useful treatment for the rapid correction of iron deficiency anaemia and can be used to avoid or reduce the requirement for allogeneic blood transfusion. Several intravenous iron preparations are available commercially which differ in cost, mode of administration and side effect profile. There are few data directly comparing the efficacy of these preparations. In this retrospective single-centre study, we present the results from two hundred and eight patients treated using three different iron preparations (iron dextran, iron sucrose and ferric carboxymaltose) and compare the effect on haemoglobin levels and other measures of iron deficiency six weeks after treatment. Within the limitations of our study design, we show a statistically and clinically significant difference in efficacy between these preparations.

## 1. Introduction

Intravenous iron can be a useful treatment for iron-deficiency anaemia in several clinical situations, including in patients who are intolerant to or unresponsive to oral iron [1], patients undergoing elective surgery [2], and in patients in whom the severity of the anaemia requires rapid correction [3]. Intravenous iron therapy may reduce the requirement for allogeneic blood transfusion [4]. Iron deficiency is the most common cause of anaemia worldwide and in the United Kingdom; “Better Blood Transfusion” guidelines require hospitals to provide alternatives to allogeneic blood transfusion where possible, and the use of intravenous iron may be an effective way to achieve this.

At the time of writing, there were three commercially available forms of intravenous iron in use in the United Kingdom. Iron sucrose (IS, Venofer, Vifor Pharma) is administered as an intravenous infusion containing 200 mg of iron over two hours, and subsequent doses may be given at 48-hour intervals until the desired dose of iron has been achieved. Iron dextran (ID, CosmoFer, Vitaline Pharma) is given as an intravenous infusion containing any dose up to 2000 mg of iron depending upon the patients' calculated iron deficit; the rate of infusion is titrated according to patient tolerance, and, for example, a 1000 mg dose would usually be infused over a total of 5 hours. Ferric carboxymaltose (FCM, Ferinject, Vifor Pharma) has become available recently and is administered as an intravenous bolus (containing 500 mg elemental iron) or intravenous infusion over 30 minutes (containing 1 g of elemental iron) (source: British National Formulary).

Although FCM has been shown to be safe and effective in the treatment of iron-deficiency anaemia [5], at present there are no data comparing it to existing preparations. Furthermore, although the costs of ID and IS are comparable, FCM is significantly more expensive (basic NHS prices for 1 g of iron: iron sucrose *£*70.80, iron dextran *£*79.70, of ferric carboxymaltose *£*217.50) (British National Formulary).

In addition to the drug cost, factors that need to be taken into account when selecting an intravenous iron preparation include the number of hospital visits required, the administration costs (e.g., staff time, bed occupancy time), and whether any benefits will be achieved by reducing pressure on ambulatory care facilities (e.g., ability to increase other treatments).

Comparative efficacy data would therefore be useful in deciding which preparation to use in a given clinical setting. Although this should be in the form of a randomised trial, we have been able to study a large number of patients treated with these preparations through our haematology service.

## 2. Patients and Methods

We performed a retrospective analysis of all patients who had been treated in our service for iron-deficiency anaemia over a two-year period. During that period, all three preparations had been in use at different times because the supply of both ID and IS had been interrupted at times due to manufacturing problems, and FCM was introduced onto our local formulary part of the way through the period of our study.

Patients were referred to our service by their general practitioners or by other medical or surgical teams within the same hospital for assessment and management of anaemia. After clinical assessment by a haematology doctor or clinical nurse specialist, patients underwent intravenous iron treatment if they had proven iron-deficiency anaemia and were either intolerant to or unresponsive to oral iron treatment, severely anaemic to the extent that it was felt by a consultant haematologist that oral iron treatment alone would be insufficient treatment, or if they had elective surgery scheduled and required a rapid correction of their anaemia. Treatment was delivered by a nurse-led service initiative. Patients were not treated in our service if they were under the age of 16 years or if they had chronic kidney disease stage 4 or worse (eGFR <  29 mL/min—these patients were managed in a separate renal anaemia service). The mean age of the patients was 39 years with a range of 22–75 years.

In parallel to iron therapy, patients also underwent investigations for the cause of iron deficiency as was clinically appropriate in each case. All patients were stable with no active bleeding. The choice of iron therapy was based on which iron preparation was available in our unit at the time patients presented for treatment, and where more than one preparation was available, choice was by clinician's preference.

Eighty-four patients (72 female) were given iron sucrose. After a 25 mg test dose on the first infusion only, this was given at a dose of 200 mg by intravenous infusion over two hours diluted in 100 mL of normal saline, repeated a variable number of times over the following 7–14 days depending on the severity of the anaemia (although there was no set formula for this). The median number of infusions was 4, and the range was 3–6.

Forty-two patients (35 female) were given iron dextran at a dose of 20 mg/kg by intravenous infusion. After an initial test dose of 25 mg, the remainder of the dose was given in 500 mL of normal saline over 4–6 hours.

Eighty-two patients (seventy female) were treated with ferric carboxymaltose at a dose of either 500 mg by slow intravenous bolus injection (patients below 60 kg body weight) or 1 g diluted in 500 mL of normal saline and infused over 30 minutes (patients above 60 kg).

Six weeks after intravenous iron treatment, patients were reviewed and had a blood sample taken for measurement of the full blood count and serum ferritin.

## 3. Results

**Figure 1:**
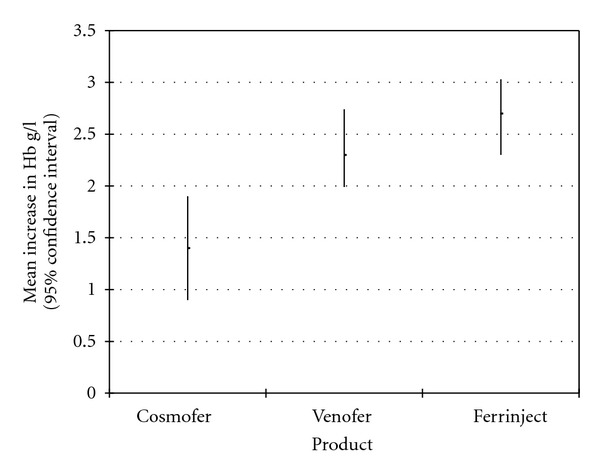
Mean increase in Hb six weeks after intravenous iron treatment with each product.

The three groups of patients were similar with respect to age, gender, baseline haemoglobin level, baseline red cell indices, and serum ferritin (see Table 1). At six weeks after infusion (or six weeks after the last infusion in patients who received IS), the mean haemoglobin had risen from baseline significantly in all three groups. The mean (± standard deviation) increase in haemoglobin level from baseline was 1.4 g/dL (0.9–1.9) with ID, 2.4 g/dL (1.99–2.74) with IS, and 2.7 g/dL (2.30–3.03) with FCM (see Figure 1).

When we compared these three groups using Student's *t*-test, we found that the increase in haemoglobin concentration was significantly greater in both IS and FCM compared with ID (*P* = 0.04 and <0.01, resp.). However, there was no statistically significant difference between the groups treated with IS and FCM. 

There was a significant increase in both mean serum ferritin concentration and mean MCV after treatment in all groups (see Table 2); however, we were unable to show a statistically significant difference in these variables between the groups.

Two patients experienced an adverse event attributable to intravenous iron therapy. One patient in the ID group experienced hypotension and a rash during infusion, and one patient in the FCM group noticed an urticarial rash shortly after the infusion.

## 4. Discussion

There are several limitations to our study. First, the doses of elemental iron received by the three treatment groups were different. However, the dosing system we used and therefore our results reflect the way in which these preparations are typically used in clinical practice. Furthermore, this factor would have biased our results in favour of iron dextran because patients usually received a higher dose of elemental iron if they were given ID (any patient who weighed over 50 kg would have received more than 1 g of iron in the ID group), and yet our results still show a significantly greater increase in Hb with FCM and IS compared with ID. Second, as we took our followup sample for full blood count six weeks after the last infusion, and patients treated with IS typically received 4–6 infusions over a two-week period, theoretically our results would have been biased in favour of IS because the patients in this treatment group would have had a slightly longer time for the earlier doses of iron to take effect.

In spite of these two major limitations, both of which would have biased our results against FCM, we saw increases in haemoglobin level that were significantly greater than in patients treated with ID and equivalent to those treated with IS. The results of cost versus benefit analysis will vary in different settings, but in many cases, we suggest that the higher cost of FCM may well be offset by savings in staff time and bed space (especially compared with IS: one infusion compared with 4–6) and with greater efficacy (especially compared with ID).

Our study was much too small to assess differences in adverse effects; however, ID has been reported previously to have a higher rate of anaphylaxis compared with newer preparations [6].

As this is a retrospective study with several limitations, our observations will require verification, preferably with a randomised controlled trial.

## Figures and Tables

**Table 1:** Baseline characteristics.

Preparation	Iron	Iron	Ferric
	dextran	sucrose	carboxymaltose

Number of patients	44	84	82
% Female	84	85	85
Mean haemoglobin	9.7	9.3	9.0
Median (SD)	9.6 (1.37)	9.5 (1.8)	9.0 (1.75)
haemoglobin			
Mean MCV	79	78	76
Median (SD) MCV	79 (9.51)	79 (14.4)	75 (10.8)
Mean ferritin	23	14	27
Median (SD) ferritin	9 (36.9)	11 (42.4)	27 (52.9)

**Table 2:** Increase from baseline in Hb, Ferritin, and MCV in each group.

Preparation	Iron dextran	Iron sucrose	Ferric carboxymaltose
Mean (± SD) haemoglobin increase g/dL	1.4 (0.9–1.9)	2.4 (1.99–2.74)	2.7 (2.30–3.03)
Mean (± SD) MCV increase (fl)	5.8 (4.0–7.6)	5.6 (2.9–8.3)	7.0 (4.6–9.7)
Mean (± SD) ferritin increase (mcg/dL)	149 (93–205)	109 (84–133)	149 (99–200)
